# Diseases of the Nervous System

**Published:** 1905-06-03

**Authors:** 


### DISEASES OF THE NERVOUS SYSTEM.

(*Continued from page* 155.)

**Syphilis of the Nervous System.**—In a clinical lecture on the effects of syphilis in the nervous system Turner[5] said that it is best to classify them into "vascular" and "degenerative"—the vascular lesions being endarteritis, periarteritis, and gummata, whilst the degenerative form is the pathological basis of tabes and general paresis. A time limit is unsatisfactory, and it is impossible to call the vascular lesions "secondary" and the degenerative lesions "tertiary," for the latter may come on five years after infection, and the former ten years after, There are no absolutely pathognomic signs of a syphilitic lesion, but many highly suggestive of it. Thus, there may be an association or succession of symptoms suggesting a double or multiple lesion. Again, a syphilitic lesion is most probably subacute in its onset and subchronic in its course. It does not present the features of the sudden myelitis due to bacterial causes, nor the character of the chronic degenerations such as are seen in disseminat ed sclerosis. A third characteristic feature is remission and relapse of the symptoms. A fourth is variability and uncertainty in the results of treatment. Many cases react readily to iodide and mercury, but quite a number do not. A characteristic prodromal symptom of syphilitic lesions is headache of a severe nature coming on in the late afternoon and preventing sleep from its intensity. A neurasthenic headache is always worse in the morning. With this headache there is sometimes found mental apathy and torpor. As soon as the paralysis appears, the headache, insomnia, and mental apathy disappear. The symptom which distinguishes a gummatous lesion of the cortex from a vascular one is the occurrence of localised convulsions or Jacksonian epilepsy. A gumma situated subcortically does not cause convulsions—only paralysis. The third form of syphilitic lesion is meningo-encephalitis, or meningo-myelitis, a gummatous small-celled infiltration affecting the pia-arachnoid and subjacent nervous tissue. In the case of the spinal cord there may be symptoms denoting a transverse lesion. In some cases the Brown-Séquard symptom is present—loss of motion of one lower limb and loss of sensation in the opposite limb. This clinical combination is due to thrombotic lesions involving one side of the cord—generally due to syphilis, or to tumour formation within the cord. Prognosis in cerebro-spinal syphilis is not very satisfactory—only one-third of the cases are cured; a small percentage, some 10 or 12, die; and the others are crippled for the rest of their lives. The most satisfactory form to treat is Jacksonian epilepsy, or cortical meningo-encephalitis; after this come basal lesions. The least favourable are the vascular lesions, or those hemiplegias arising from thrombosis with subsequent softening.

**Reflexes in Hysteria.**—Crocq,[6] from a study of 100 cases of hysteria, draws the following conclusions: Abolition of the pharyngeal reflex is common, especially in the forms accompanied by anæsthesia, but is of little diagnostic value, as it is found in many other affections, and even normally. Exaggeration of the tendon reflexes is frequent, especially in hysterical seizures, but, being found in many toxic conditions, and even normally, is not of any pathognomic value. Abolition of plantar sensibility is frequent. Simultaneous abolition of cortical plantar reflex or flexor type and of medullary or fascialata type is very frequent; as these two reflexes are remarkably constant in the normal state, their abolition is of considerable importance in the diagnosis of hysteria. Anæsthesia may or may not be present in cases of abolition of these reflexes. Ankle clonus is not very rare, patellar clonus less frequent, and wrist clonus was not seen. Ankle and patellar clonus are especially frequent in the paralytic forms. A Babinski reflex is never present in typical cases. The abdominal reflex shows inconstant variations—it may be absent, feeble, or exaggerated.

**Veronal** has been employed as a hypnotic on a large number of patients by Pisarski.[7] The dose given varied from 4 to 45 grains; one of 15 grains as a rule caused sleep in half an hour and lasted from six to seven hours. In three-quarters of the cases there were no unpleasant after-effects; in a quarter transitory headache, tinnitus, or unsteadiness of gait appeared, and this happened only when large doses had been given. The drug was found to have a slightly cumulative action. The effect is mainly a sedative one, and it has little power of soothing pain or allaying cough. No change in the temperature or appetite occurred, and the pulse tension was never heightened.

[5] Clin. Jour., Dec. 21, 1904. [6] Brit. Med. Jour., Epitome, Dec. 24, 1904. [7] Ibid.

